# Catalpol protects synaptic proteins from beta-amyloid induced neuron injury and improves cognitive functions in aged rats

**DOI:** 10.18632/oncotarget.17951

**Published:** 2017-05-17

**Authors:** Zhiming Xia, Fengfei Wang, Shuang Zhou, Rui Zhang, Fushun Wang, Jason H. Huang, Erxi Wu, Yongfang Zhang, Yaer Hu

**Affiliations:** ^1^ Research Laboratory of Cell Regulation, School of Medicine, Shanghai Jiaotong University, Shanghai 200025, China; ^2^ Department of Neurosurgery, Baylor Scott & White Health, Temple, Texas 76508, USA; ^3^ Department of Neurology, Baylor Scott & White Health, Temple, Texas 78508, USA; ^4^ Department of Surgery, Texas A & M University College of Medicine, Temple, Texas 76504, USA; ^5^ Department of Psychology, Nanjing University of Chinese Medicine, Nanjing, Jiangsu 210023, China; ^6^ Department of Pharmaceutical Sciences, Texas A & M University College of Pharmacy, College Station, Texas 77843, USA; ^7^ Current address: Department of Nuclear Medicine, Shandong Provincial Hospital, Shandong University, Jinan, Shandong 250021, China

**Keywords:** catalpol, synaptic proteins, neuron injury, cognitive functions, Alzheimer’s disease, Gerotarget

## Abstract

Synapse loss is one of the common factors contributing to cognitive disorders, such as Alzheimer’s disease (AD), which is manifested by the impairment of basic cognitive functions including memory processing, perception, problem solving, and language. The current therapies for patients with cognitive disorders are mainly palliative; thus, regimens preventing and/or delaying dementia progression are urgently needed. In this study, we evaluated the effects of catalpol, isolated from traditional Chinese medicine *Rehmannia glutinosa,* on synaptic plasticity in aged rat models. We found that catalpol markedly improved the cognitive function of aged male Sprague-Dawley rats and simultaneously increased the expression of synaptic proteins (dynamin 1, PSD-95, and synaptophysin) in the cerebral cortex and hippocampus, respectively. In beta-amyloid (Aβ) injured primary rat’s cortical neuron, catalpol did not increase the viability of neuron but extended the length of microtubule-associated protein 2 (MAP-2) positive neurites and reversed the suppressive effects on expression of synaptic proteins induced by Aβ. Additionally, the effects of catalpol on stimulating the growth of MAP-2 positive neurites and the expression of synaptic proteins were diminished by a PKC inhibitor, bisindolylmaleimide I, suggesting that PKC may be implicated in catalpol’s function of preventing the neurodegeneration induced by Aβ. Altogether, our study indicates that catalpol could be a potential disease-modifying drug for cognitive disorders such as AD.

## INTRODUCTION

Synaptic loss is strongly correlated with cognition impairment [1] and is considered as a major neurobiological abnormality causing cognitive dysfunctions [2]. Importantly, synaptic failure is an early event in the pathogenesis of Alzheimer’s disease (AD) and is clearly detectable in patients with mild cognitive impairment (MCI), a prodromal state of AD [3, 4]. Individuals with mild AD display a 55% decrease of total synapse number in hippocampal CA1 subfield, whereas individuals with MCI have an 18% decline in synaptic contacts in the same region [5]. The loss of synapses is not only resulted from the death of neurons but also caused by the dysfunction of synaptic connections between certain groups of neurons. Callahan *et al.* [6] reported a 50% reduction of synaptophysin mRNA level over CA1 pyramidal neurons containing neurofibrillary tangles (NFT) relative to near neighbor NFT-free neurons in AD hippocampus. Moreover, there is a graded and progressive decrease of synaptophysin mRNA level as AD progresses in each individual neuron [7].

Synapse function depends on the coordination of a molecular complex involved in transmitter systems, second messenger systems, mitochondria, local protein synthesis, vesicle trafficking and other functions. Although the loss of synapses leads to disruption of the neuronal circuitry in AD, an increasing body of evidence suggests that synapses can be damaged in their functions even before structural deterioration [8-14]. Yao *et al.* [9] found that a group of genes involved in synaptic vesicle (SV) trafficking was down-regulated in the frontal cortex of AD patients, whereas several synaptic genes in other aspects of the synaptic structure and function remained unchanged. Sze *et al.* [10] observed that the expression of presynaptic vesicle proteins, instead of synaptic plasma membrane proteins, were selectively decreased in AD brain, indicating that there are certain defects in synthesizing these proteins or abnormal metabolism of these proteins in AD. Shimohama *et al.* [11] reported that contents of vesicle proteins were decreased more dramatically than those of presynaptic plasma membrane proteins in AD. These results suggest that dysregulated synaptic function and ineffective neurotransmission may be a mechanism of cognitive decline.

Catalpol, an iridoid glycoside isolated from the fresh *Radix rehmanniae*, is an effective compound against neurodegenerative diseases including AD and Parkinson’s diseases [15-20]. It can regulate cholinergic nerve system function through the effect on choline acetyl-transferase [21] and it is capable to penetrate the blood-brain barrier [22]. Our previous studies have demonstrated that the neuroprotective effects of catalpol are mainly through up-regulation of the expression of brain-derived neurotrophic factor (BDNF) [23], which is a major mediator for synaptic transmission, synapse development and plasticity in the nervous system [24, 25]. Recently, Liu *et al.* [26] reported that catalpol could increase the expression of synaptophysin, a presynaptic protein, and up-regulate the expression of PKC and BDNF in the hippocampi of aged rats. The results indicate that catalpol can modulate the synaptic plasticity of aged rats. However, it remains to be determined if catalpol regulates the expression and the function of other important synaptic proteins involved in synaptic plasticity.

Similar to humans, aged rodents exhibit age-related decline in cognitive function [27]. Among the numerous behavioral tests for assessing learning and memory, the novel object recognition test is a widely used approach for investigating recognition memory in rodents [28]. In the present study, we first tested the efficacy of catalpol on rats’ cognitive function. We then used a previously well characterized Aβ induced brain injury as a model to examine the effects of catalpol on synaptic plasticity and the effects of catalpol on the expressions of some critical molecules for cognitive function located in presynaptic and postsynaptic terminals, e.g., dynamin 1, PSD-95, synaptotagmin, and synaptophysin, which exert distinct actions on synaptic structure and function. This study elucidates the potential mechanism of catalpol in the amelioration of neurodegenerative changes and provides evidence that catalpol can be a potential therapeutic or preventive drug for cognitive disorders such as AD.

## RESULTS

### Catalpol ameliorates the performance of aged rats without affecting Aβ_25-35_ injured neuron on survival

**Figure 1:**
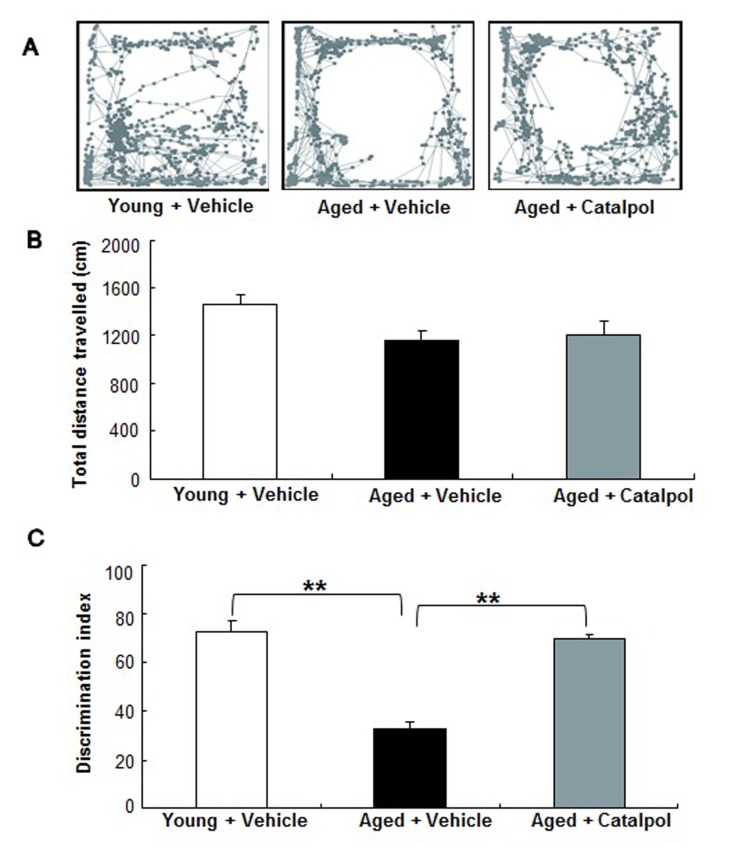
Effects of catalpol (10 µM) on the locomotor activity and cognitive function of aged SD rats **A.** Representative traces of different groups in an open field test. **B.** Statistical analysis of the total distance travelled by different groups in the open field test. **C.** Statistical comparison of the discrimination index in novel object recognition test. *n* = 8 animals in each group. All values are presented as the mean ± SEM. ** indicates *p* < 0.01, compared with the aged group (Aged + Vehicle).

Our previous study demonstrated that catalpol protected primary cortical neurons against Aβ injury [23]; however, it remains unclear whether catalpol can improve the performance of aged rats. In this study, to determine the effects of catalpol on rats’ cognitive function, rats were administrated with either capitol or vehicle. After two months of administration of catalpol or vehicle, the locomotor activity of the rats was estimated using an open field test. As shown in Figure 1A and 1B, there was no significant difference in the distance travelled among the three groups (Young+Vehicle: 1467±80 cm; Aged+Vehicle: 1153±87 cm; Aged+Catalpol: 1211±108 cm; p>0.05). A novel object recognition test was also performed to examine the effect of catalpol on the memory of aged rats. During the retention test, no significant difference in the total exploration time of the two objects was observed (total exploration time of both objects: Young+Vehicle: 18.6±5.1 s; Aged+Vehicle: 15.5±1.9 s; Aged+Catalpol: 14.5±2.8 s; p>0.05; n=8/group). However, when analyzing the travel pattern of the rats using the discrimination index, we observed significantly more time for the catalpol-treated aged rats with the novel object than the aged rats treated with vehicle (Figure 1, p<0.05). This indicates that the catalpol improved the working memory performance of the aged rats after 2 months of administration. As aged rats treated with catalpol were indistinguishable from the young rats in this test, we conclude that chronic catalpol treatment ameliorated the impaired performance of aged rats.

We then examined whether catalpol is able to rescue damaged neurons. We thus analyzed the effects of catalpol on Aβ_25-35_ ( the active region of full-length peptides [29]) induced neuron toxicity on neuron survival. Based on the results of neuron survival assay using MTT, Aβ_25-35_ has significant neuron toxicity at concentration of 10 μM (Supplementary Figure 1). We thus determined the effects of different concentrations of catalpol on 10 μM of Aβ_25-35_ to induce neuron injury for various time periods and found that there is no ameliorating effect of catalpol on cell survival of the primary cortical neurons injured by Aβ_25-35_ (Supplementary Figure 1B-C).

### Effects of catalpol on synaptic protein expressions in the cerebral cortex and hippocampus of aged rats

**Figure 2:**
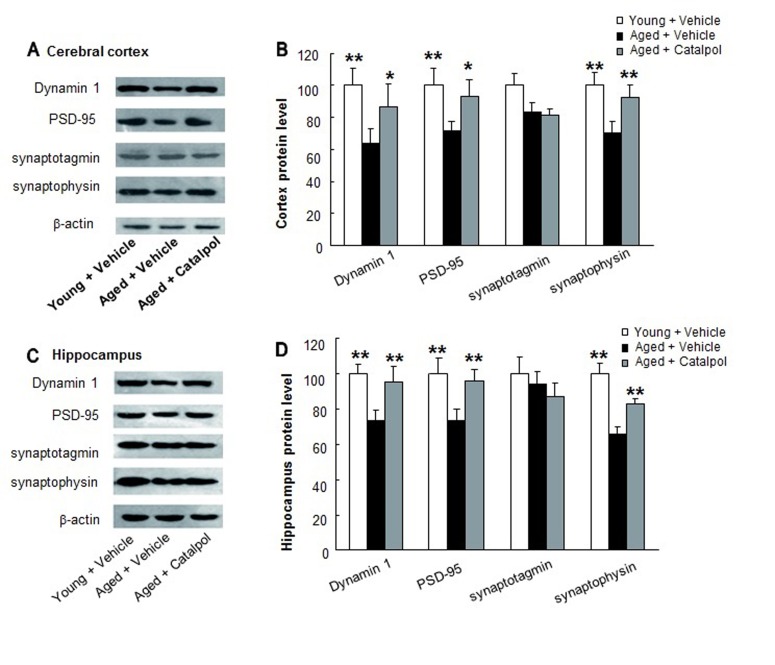
Effects of catalpol (10 µM) on the expression of synaptic proteins in cerebral cortex and hippocampus **A.** and **C.** The representative images of Western blots for synaptic proteins in cerebral cortex and hippocampus. β-actin is used as the internal control. **B.** and **D.** Statistical comparisons of integrated optical density (IOD) in each group. All values are normalized to the young group and presented as the mean ± SEM, *n* = 5. * and ** indicate p<0.05 and p<0.01, respectively, compared with the group (Aged + Vehicle).

Synaptic proteins play important roles in neuronal development and synaptic plasticity. Dynamin 1 is a neuron-specific GTPase that is responsible for endocytic vesicle fission from the synaptic terminal membrane [30]. PSD-95, a synaptic scaffolding protein, plays a critical role in synaptogenesis, receptor clustering, and the modulation of synaptic plasticity [31]. The expression levels of PSD-95 in the brain of mice is positively correlated with learning and memory [14] and it is regarded as a synaptic marker [32]. Synaptotagmin harbors two Ca^2+^-binding domains (C2A and C2B), which serve as the major Ca^2+^ sensor for triggering membrane fusion [33]. Synaptophysin, one of the most abundant integral proteins of the SV membrane, interacts with other synaptic proteins and participates in several steps of synaptic function [34]. To elucidate the mechanism of catalpol’s effects, we further examined the effects of catalpol on the expressions of those synaptic proteins important for synaptic structure and function. Our data show that the synaptic proteins dynamin 1, PSD-95, and synaptophysin were significantly decreased in the cerebral cortex and hippocampus of aged rats, whereas synaptotagmin was decreased in cerebral cortex but not in hippocampus of aged rats compared with young rats (Figure 2). After 2 months of treatment, catalpol significantly increased the expression of dynamin 1, PSD-95, and synaptophysin in the cerebral cortex and hippocampus of aged rats, respectively. However, the expression of synaptotagmin did not change visibly after administration of catalpol (Figure 2).

### Catalpol promotes neuron recovery and enhances the expression of synaptic proteins of MAP-2 positive neurites of Aβ_25-35_ injured neurons

**Figure 3:**
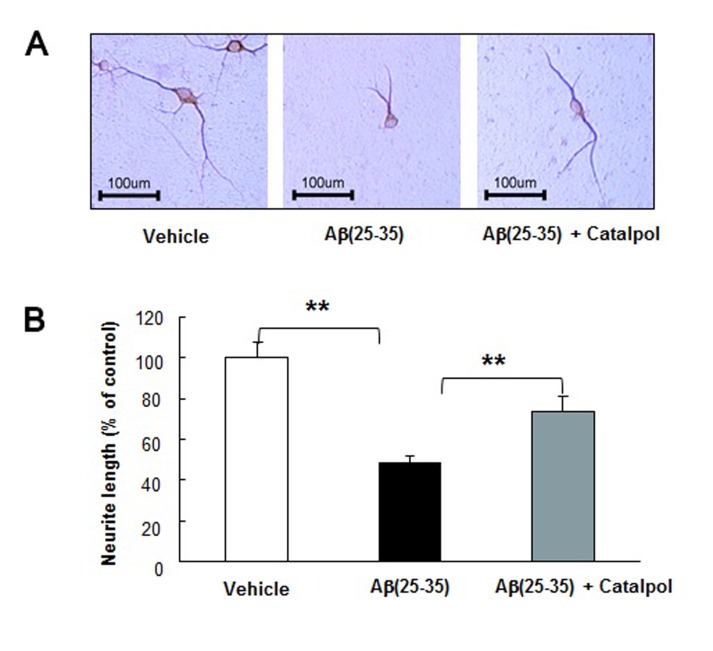
Catalpol (10 µM) restores MAP-2-positive neurite length of Aβ_25-35_-treated primary cortical neurons **A.** Representative images of immunocytochemistry staining at ×200 magnification, scale bar represents 100 μm. **B.** Statistical analysis of neurite length in various groups. Data are presented as the mean ± SEM of three independent experiments. ** indicates *p* < 0.01, when compared with the Aβ_25-35_ treated group.

To further access the efficacy of catalpol on neuron recovery, we treated neuron with Aβ_25-35_ in the presence or absence of catalpol, and then analyzed neuron growth. As shown in Figure 3, Aβ_25-35_ significantly decreased the length of MAP-2-positive neurites, compared with that of neurons treated with vehicle alone. When Aβ_25-35_ injured neurons were treated with catalpol over 48 h, the inhibitory effects of Aβ_25-35_ on the growth of MAP-2 positive neurites were partially recovered (Figure 3). The results suggest that catalpol partially reversed the neurite injury induced by Aβ_25-35_.

**Figure 4:**
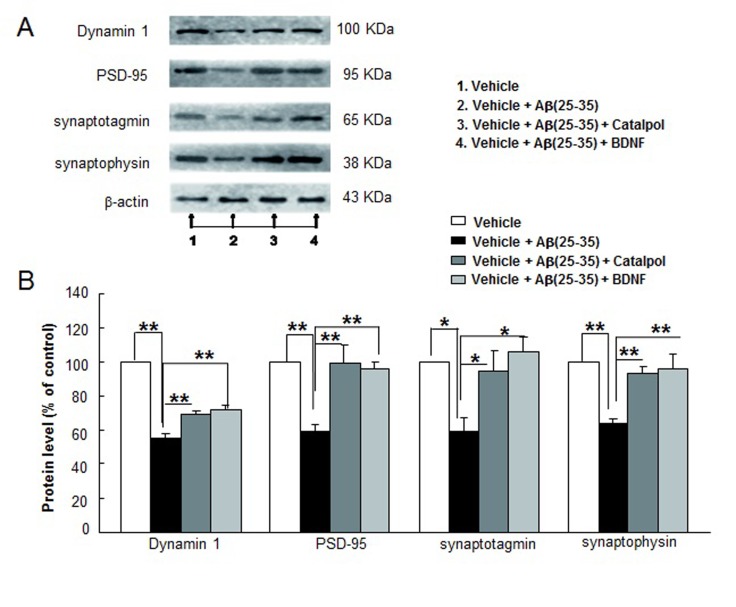
Catalpol (10 µM) and BDNF (1.85 nM) enhance synaptic protein expressions of Aβ_25-35_-treated primary cortical neurons Cortical neurons were cultured for 21 days, and then Aβ_25-35_ was added. After 12 h of Aβ_25-35_ injury, catalpol and BDNF were added to culture for 48 h following synaptic protein detection. **A.** Representative images of Western blot for synaptic protein expressions in primary cortical neurons with different treatments. β-actin is used as the internal control. **B.** Statistical comparisons of IOD in each group. All values are normalized to the control group value and expressed as the mean ± SEM of four independent experiments. * and ** indicate *p* < 0.05 and *p* < 0.01, respectively, compared with the Aβ_25-35_ treated alone group.

A body of evidence suggests that Aβ can induce abnormal expressions of some synaptic proteins [35-38]. We thus also determined the expression levels of dynamin 1, PSD-95, synaptotagmin, and synaptophysin in the neuron treated with Aβ_25-35_ in the presence or absence of catalpol. Our results show that the expression levels of dynamin 1, PSD-95, synaptotagmin, and synaptophysin in the cultured neurons were significantly decreased by Aβ_25-35_ (Figure 4). After 48 h of catalpol treatment, the expression levels of dynamin 1, PSD-95, synaptotagmin, and synaptophysin in the cells were enhanced by 25%, 69%, 59% and 45%, respectively (Figure 4). Similarly, BDNF also increased the expressions of those synaptic proteins, which was consistent with a previous report [39].

Although Aβ_25-35_ displays neurotoxic and aggregation properties similar to full-length Aβ (i.e., Aβ_1-40_ and Aβ_1-42_) and is recognized as the active region of full-length peptides, Aβ_1-42_ is the predominant peptide found in extracellular plaque deposition [29]. We thus performed a simple comparison of neurotoxicity between Aβ_1-42_ and Aβ_25-35_ on cultured rat’s neurons using MTT assay and the reversed peptide Aβ_42-1_ served as a control. As shown in the Supplementary Figure 2, the cell viability of neuron culture after treated with 5 μM Aβ_1-42_ for 12 h was similar to that of the neuron culture treated with 10 μM Aβ_25-35_ while the reverse peptide Aβ_42-1_ did not affect neuron survival. Therefore, Aβ_1-42_ was selected for further studies and 5 μM Aβ_1-42_ was chosen to further evaluate the neuroprotective effects of catalpol on primary cultured neurons.

### Bisindolylmaleimide I (Bis) abolishes catalpol’s protective effects on synaptic proteins and MAP-2-positive neurite growth in Aβ_1-42_ injured neuron

**Figure 5:**
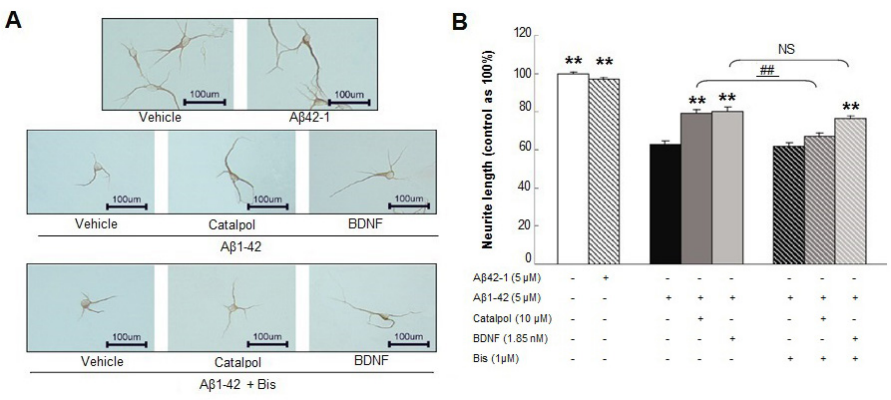
The effects of catalpol (10 µM) and BDNF (1.85 nM) on the growth of MAP-2-positive neurite treated with Aβ_1-42_ in the presence or absence of Bis **A.** Representative images of staining at ×200 magnification; the scale bar represents 100 μm. **B.** Statistical analysis of neurite length in various groups. Data are presented as the mean ± SEM of three independent experiments. ** indicates *p* < 0.01, when compared with the Aβ_1-42_ treated alone group. ^##^ indicates *p* < 0.01, when compared with the corresponding group without Bis treatment. NS indicates no significant difference.

To further determine the possible underlying mechanism of catalpol’s protective role on neurons, we treated cultured neurons with Aβ_1-42_ or Aβ_1-42_ or vehicle control in the presence of catalpol and/or BDNF and/or Bis. As shown in Figure 5, Aβ_1-42_ shortened the length of MAP-2-positive neurites, whereas the reverse peptide Aβ_42-1_ had no effect on the neurite outgrowth of neurons. Catalpol and BDNF also significantly extended the MAP-2-positive neurite length of Aβ_1-42_ injured neurons. Considering the important role of PKC signaling in neurite outgrowth, we examined whether PKC pathway was involved in the effects of catalpol and BDNF on neurite outgrowth. After inhibition of PKC activity by Bis, the effect of catalpol on neurite outgrowth was completely abolished, i.e., the length of the neurons treated with catalpol + Bis + Aβ_1-42_ displayed no difference compared with that of the neurons treated with Aβ_1-42_ + Bis and decreased significantly in comparison with that of the neurons treated with catalpol + Aβ_1-42_. However, the effect of BDNF on neurite outgrowth was not significantly influenced by the Bis treatment (Figure 5).

**Figure 6:**
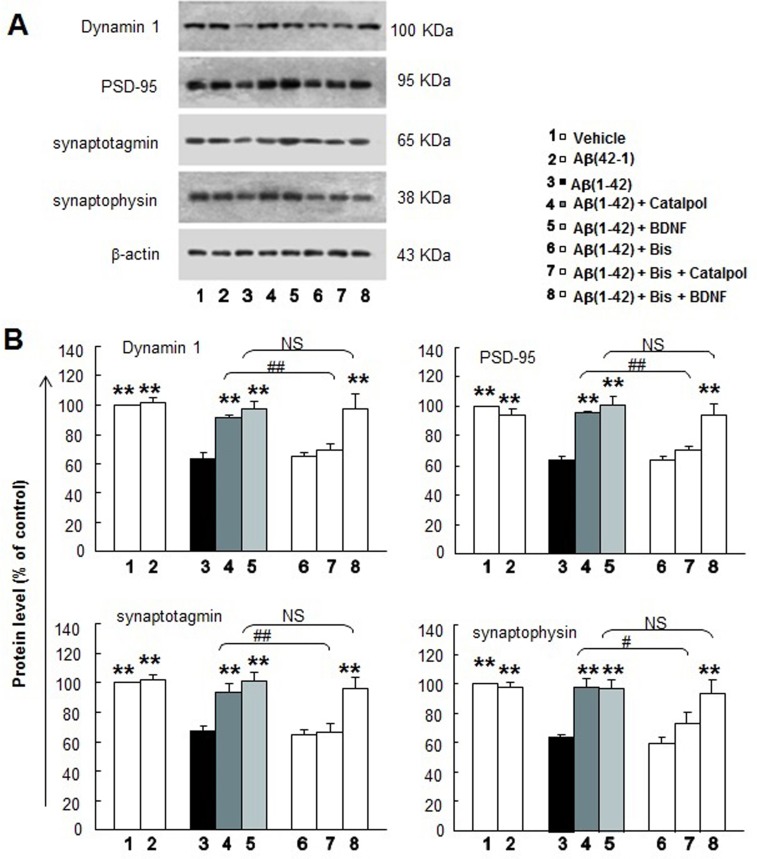
The effects of catalpol (10 µM) and BDNF (1.85 nM) on synaptic protein expression of Aβ_1-42_-treated cortical neurons with and without Bis treatment **A.** Representative images of Western blots for synaptic protein expressions in primary cortical neuron with different treatments. β-actin is used as the internal control. **B.** Statistical comparisons of IOD in each group from (A). All values are normalized to the control group and presented as the mean ± SEM of four independent experiments. ** indicates *p* < 0.01, when compared with the Aβ_1-42_-treated alone group. ^##^ indicates *p* < 0.01, when compared with the corresponding group without Bis treatment. NS indicates no significant difference.

To further determine the mechanism of catalpol in protecting effects on neurons, we examined the effects of catalpol on the expression levels of dynamin 1, PSD-95, synaptotagmin, and synaptophysin in Aβ_1-42_ injured neurons with or without the Bis treatment. As shown in Figure 6, Aβ_1-42_ induced the decreases of dynamin 1, PSD-95, synaptotagmin, and synaptophysin, while reverse peptide Aβ_42-1_ did not affect the expression of those synaptic proteins. Catalpol and BDNF also significantly increased the expression of those proteins to varying degrees. Interestingly, catalpol did not enhance the expression of those synaptic proteins after Bis treatment. Similar to the effect on neurite outgrowth, BDNF did not attenuate the effect on synaptic proteins after Bis treatment.

## DISCUSSION

In this study, by using aged male SD rat and Aβ injured primary rat cortical neuron models, we observed that aged rats display a significant impairment in object-recognition memory, as reflected by discrimination index. We then examined the protective effects of catalpol on neurons. We found that catalpol markedly improved the cognitive function of aged rats and enhanced the expression of synaptic proteins in both the cerebral cortex and hippocampus. Although it did not improve the cell viability of Aβ injured neuron cells, we observed that catalpol restored the length of MAP-2 positive neurites and the expression of synaptic proteins in Aβ injured primary rat cortical neurons. Our study reveals the protective effects of catalpol against synaptic impairment and suggests that catalpol could be used as a disease-modifying drug for cognitive disorders such as AD.

Aβ has been considered as a leading causative agent inducing AD; however, the precise toxic mechanisms of Aβ remain blurry. Recent studies specifically emphasize the pathogenic role of Aβ at the synapse [40, 41]. In our *in vitro* study, we observed the effects of catalpol on the synaptic plasticity in Aβ damaged neural culture. As shown in Figures 3-5, catalpol does not prevent the death of Aβ_25-35_-injured neurons but extends the MAP-2-positive neurite length and reverses the decrease of synaptic proteins of surviving neurons. The reversing effect of catalpol on synaptic proteins could rival that of BDNF, which is essential for the development of the central nervous system and for neuronal plasticity [42].

Although many studies were performed to locate the specific region for the object recognition [43-46], the specific brain areas involved in object recognition remain unclear. For instance, two groups attempted to disrupt the hippocampus of rodents to reproduce the recognition memory deficits in humans and primates have yielded mixed results in the object recognition test [47, 48]. The perirhinal cortex, located dorsally to the hippocampal formation, is considered to be involved in object recognition tests [47]. In this study, we dissected the cerebral cortex and hippocampus of rats after the behavioral test to determine the contents of some synaptic proteins.

Recent morphological studies have revealed that the neuron number is preserved in normal aging, even in aged rats with learning deficits [49]. This implies that cognitive declines with aging are due to synapse loss and/or synaptic dysfunction rather than neuronal losses. In the present study, we found that the synaptic proteins dynamin 1, PSD-95, and synaptophysin were decreased, respectively, in the cerebral cortex and hippocampus of aged rats, whereas synaptotagmin was decreased in the cerebral cortex but not in the hippocampus of aged rats (Figure 2). The results are in accordance with the majority of previous reports [50, 51]. However, Nicolle *et al.* [52] reported no loss of synaptic proteins in the hippocampus of aged Long Evans rats, including aged rats with severe cognitive impairment. The contradiction may originate from the differences in species and aging degree of aged rats, the selected brain regions and the tissue preparation. In our study, after 2 months of administration, catalpol significantly attenuated the decreased expressions of dynamin 1, PSD-95, and synaptophysin in the cerebral cortex and hippocampus of aged rats, respectively (Figure 2). These findings provided the molecular basis for catalpol’s improvement of cognition function.

Synaptic plasticity is a dynamic process necessary for both synaptogenesis and modification of existing synapses [53]. MAP-2 is essential for the development and maintenance of neuronal morphology. The disruption of MAP-2 decreases microtubule bundling and impairs dendritic elongation, leading to learning and memory deficits [54]. Catalpol, extending the MAP-2-positive neurite length of Aβ-damaged neurons, may improve the reorganization of dendritic architecture and facilitate synaptogenesis. Although we did not evaluate the effect of catalpol on the dendrite spine or synapse density with electron microscopes, the incremental expressions of synaptophysin and PSD-95, pre- and post-synaptic markers, respectively, may indirectly represent the increase of synapse density. The increases in dynamin 1 and synaptotagmin levels, two important proteins in synaptic transmission, may also indicate the effect of catalpol on the regulation of synaptic transmission. As shown in this study, we conclude that catalpol could increase synaptic plasticity and thus alleviate neural injury.

PKC signaling plays an essential role in both memory acquisition and maintenance, whereas deficits in PKC signal cascades in neurons represent one of the earliest changes in the brains of AD patients [55]. It has been demonstrated that activation of PKC increases the expression of BDNF, synaptic remodeling, and synaptogenesis in the hippocampus and related cortical areas of AD transgenic mice [55, 56]. Catalpol can increase the expression of PKC in the hippocampus of aged SD rats [26], but whether PKC signaling is involved in the effects of catalpol on neural repair should be clarified. Surprisingly, the effects of catalpol on neurite length and expression of synaptic proteins are fully eliminated after the treatment of the PKC inhibitor Bis, while the effects of BDNF did not change (Figures 4-5). Bis is a commonly used highly selective inhibitor of all PKC isoforms (Ki = 10 nM), [57] which is also with inhibitory effects of Glycogen Synthase Kinase 3 (GSK-3) (IC_50_=360 nM) [58]. Whether the effect of catalpol on neural repair depends on PKC signaling solely or by simultaneously regulating GSK-3 or other molecules requires further investigation.

It is worth emphasizing that synapse and dendritic spine pathology are the primary features of AD, prior to subsequent neuron loss. Interventions that protect synapses would be more effective for preventing or delaying the progression of AD, as the synaptic and spine pathology are closely related to recognition decline [40]. Catalpol should be an appropriate intervention for rescuing synaptic pathology by increasing the expressions of synaptic proteins. Thus, the results from this study provides evidence that catalpol could be regarded as a potential new disease-modifying drug for treatments of cognitive disorders such as AD.

## MATERIALS AND METHODS

### Reagents

Aβ_25–35_ (Sigma, Saint Louis, MO, USA), Aβ_1–42_ (Sigma, Saint Louis, MO, USA) and Aβ_42-1_ (AnaSpec, San Jose, CA, USA) were dissolved in sterile distilled water and incubated at 37°C for 4 days for aggregation. DMEM/F12, B-27 supplement and recombinant human BDNF were obtained from Gibco BRL (Rockville, MD, USA) Trypsin, dimethylsulfoxide (DMSO), and 3-(4,5-dimethylthiazol-2-yl)-2,5-diphenyl tetrazolium bromide (MTT) were purchased from Sigma. Primary antibody mouse anti-microtubule-associated protein 2 (MAP-2), mouse anti-dynamin 1, rabbit anti-PSD-95, mouse anti-synaptotagmin and mouse anti-synaptophysin were purchased from Chemicon (Temecula, CA, USA). Mouse anti-β-actin antibody was purchased from Santa Cruz Biotechnology. Secondary antibodies were purchased from Boster (Wuhan, China). Bisindolylmaleimide I (Bis) was obtained from Merck Calbiochem (Darmstadt, Germany). Catalpol was extracted from the root of *Rehmannia glutinosa* and identified by MS and NMR in our laboratory [23]. Its purity was >97% as determined by HPLC. Catalpol was dissolved in sterile distilled water with different concentrations in the present study.

### Animal models and treatments

Male Sprague Dawley (SD) rats were obtained from the Shanghai SIPPR-BK Laboratory Animal Company. Animal experiments were performed according to the National Institute of Health Guide for the Care and Use of Laboratory Animals, and the animal protocol was approved by the Animal Ethics Committee of School of Medicine, Shanghai JiaoTong University. Aged rats (23-24 months old) were randomly divided into two groups according to body weight: a group treated with vehicle (sterile distilled water) and the other group was treated with 15 mg/kg/d catalpol. Young rats (3 months old) were treated with the vehicle as the young control. Catalpol or vehicle was administered by gavage once a day for 2 months.

### Behavioral tests

#### Open field test

Spontaneous activity of rats was examined in an open-field box (60 cm × 75 cm × 75 cm). The rats were placed into the box and allowed to freely explore for 5 min. The locomotor activity was recorded by a video camera directly above the box and analyzed offline by a video track system (Noldus Ethovision).

#### Object recognition test

The novel object recognition test, including a training and retention session, was conducted in an open-field arena. Two days before test, rats received a duration of 3-min per day habituation in the arena and room environment. During the training session, two identical novel objects were placed into the arena 15 cm away from each other, and the animals were allowed to explore for 3 min. The rats were then placed back to their home cage. One hour later, the rats were returned to the arena and exposed to one familiar object and a novel object for 3 min in the retention session. The training and retention sessions were video recorded, and the time used by each animal to explore each object was scored manually by an investigator without knowledge of the experimental groups. Object exploration was defined as sniffing or touching the object with the nose and/or forepaws. When the animal used the object as a prop to explore the environment, this was not considered an exploration. A discrimination index (total time spent with new object/total time of object exploration) was used to measure recognition memory [59]. After the object recognition test, the animals were sacrificed, and the brain tissues were collected and stored at -70°C until use.

### Brain tissue sample preparation and Western blotting

The right brain hemisphere was weighed and suspended in lysis buffer (25 mM Tris-HCl pH 6.8, 1% SDS, 5% glycerol, and 200 mM DTT), at a ratio of 1 ml of lysis buffer per 100 mg of tissue. The suspension was sonicated for approximately 30 s and centrifuged at 12000 ×g for 5 min, and the supernatant was then transferred to a fresh tube. Total protein was determined with the Bradford Protein Assay Kit (Pierce), and samples (50 μg) were analyzed by immunoblot as described previously [60]. Briefly, the samples were separated on a 8% SDS polyacrylamide gels and transferred to a PVDF membrane, blocked in 5% skim milk, and incubated overnight at 4°C with primary antibody (mouse anti-dynamin 1 antibody, 1:1000; rabbit anti-PSD-95 antibody, 1:1000; mouse anti-synaptotagmin antibody, 1:1000; mouse anti-synaptophysin antibody, 1:1000; anti-β-actin antibody, 1:1000) followed by incubation with secondary antibody. The results were visualized using ECL reagents (Pierce) and quantified using an image analyzer (Gel Doc 2000, Bio-Rad). The values were finally normalized to β-actin. For cultured cells, after treatments, the cells were washed with cold PBS and harvested with a cell scraper and sonicated for approximately 30 s in the cell lysis buffer, and the supernatant was collected by centrifugation at 12000 ×g for 5 min at 4°C. The total protein of each sample was determined using the Bradford assay, and samples (30 μg) were analyzed by immunoblotting as mentioned above.

### Primary culture of cortical neurons and treatment

The primary cortical neurons were prepared and cultured with a previously described method [23]. Briefly, the cortexes of newborn SD rats (within 24 h after newborn) were dissected, and the meninges were removed. The cells dispersed by trypsin digestion and trituration were then re-suspended in DMEM/F12 containing 10% fetal bovine serum supplemented with 1% penicillin-streptomycin, plated on poly-L-lysine-coated multi-well plates, and incubated at 37°C in 5% CO_2_. The medium was replaced by DMEM/F12 plus B27 on the second day and then refreshed twice a week. After 12 h of 10 µM Aβ incubation, catalpol (10 µM) or BDNF (1.85 nM) was added to the cortical neuron culture. In the cases of PKC inhibitor treatment, the PKC inhibitor Bis was applied 2 h ahead of catalpol or BDNF. On day 7 of treatment, the neurons were subjected to neuronal viability assay and the assessment of protein expression using immunocytochemistry. For detection of protein levels using the Western blot, the cells were cultured for 21 days before harvesting.

### Detection for neuronal viability

Neuronal viability was assessed using MTT assay, based on the conversion of MTT into formazan crystals by living cells. After different treatments, 10 μl of MTT stock solution (5 mg of MTT in 1 ml of PBS) was added to the wells of 96-well culture plates. Following 4 h of incubation at 37°C in 5% CO_2_, the medium was fully removed, and 150 μl of DMSO was added to each well with shaking for 10 min. The absorbance of the sample was determined at 492 nm using a microplate reader (Bio-Tek ELX808), with serum-free medium as the blank. The relative cell viability (%) related to the control was calculated by [Absorbance]test/[Absorbance]control × 100%.

### Immunocytochemistry

The immunocytochemistry was performed using a previously described method [23]. Briefly, the cultured neurons were fixed with 4% paraformaldehyde and exposed to PBS (NaCl 8.5 g, Na_2_HPO_4_·12H_2_O 7.05 g, and NaH_2_PO_4_·2H_2_O 0.52 g dissolved in 1000 ml of distilled water, pH 7.4) containing 0.3% Triton X-100, followed by incubation in 5% BSA to block non-specific binding. The neurons were then incubated with a rabbit anti-MAP-2 antibody (1:1000) diluted in PBS at 4°C for overnight. After washing with PBS, the neurons were incubated with secondary antibody for 1 h at room temperature. Subsequently, the results were visualized using the ABC kit (Thermo Scientific, Grand Island, NY, USA) and analyzed using an inverted Nikon TE300 microscope at ×200 magnification. Ten areas were randomly captured from each well. The length of MAP-2 positive neurites of each neuron was measured by the NIH Image J software with the Simple Neurite Tracer plugin according to the manufacture’s instruction (http://imagej.net/Simple_Neurite_Tracer:_Step-By-Step_Instructions), whereas the neurons connecting with other neurons or with neurite endings outside the optic field were excluded. The total neurite length of all neurons counted in a group was divided by the total number of neurons, and then, the average of each group was normalized to that of the vehicle control.

### Statistical analysis

The data were analyzed using one-way ANOVA followed by a Student-Newman-Keuls *post hoc* test. All calculations were performed using the Statistical Analysis System (SAS 9.13 software). The results were shown as the mean ± SEM. The significance level was set at p<0.05.

## SUPPLEMENTARY MATERIALS FIGURES



## Abbreviations

Aβ: beta-amyloid
AD: Alzheimer’s disease
ANOVA: Analysis of variance
BDNF: brain-derived neurotrophic factor
Bis: Bisindolylmaleimide I
DMSO: dimethylsulfoxide
GSK-3: Glycogen Synthase Kinase 3
IOD: integrated optical density
MAP-2: microtubule-associated protein 2
MCI: mild cognitive impairment
MTT: 3-(4,5-dimethylthiazol-2-yl)-2,5-diphenyltetrazolium bromide
NFT: neurofibrillary tangles
NIH: National Institutes of Health
PBS: Phosphate-buffered saline
PKC: Protein kinase C
SD: Sprague Dawley
SEM: standard error of the mean
SV: synaptic vesicle

## References

[1] Terry RD, Masliah E, Salmon DP, Butters N, DeTeresa R, Hill R, Hansen LA, Katzman R (1991). Physical basis of cognitive alterations in Alzheimer’s disease: synapse loss is the major correlate of cognitive impairment. Ann Neurol.

[2] Arendt T (2009). Synaptic degeneration in Alzheimer’s disease. Acta Neuropathol.

[3] Scheff SW, Price DA, Schmitt FA, Mufson EJ (2006). Hippocampal synaptic loss in early Alzheimer’s disease and mild cognitive impairment. Neurobiol Aging.

[4] Robinson JL, Molina-Porcel L, Corrada MM, Raible K, Lee EB, Lee VM, Kawas CH, Trojanowski JQ (2014). Perforant path synaptic loss correlates with cognitive impairment and Alzheimer’s disease in the oldest-old. Brain.

[5] Scheff SW, Price DA, Schmitt FA, DeKosky ST, Mufson EJ (2007). Synaptic alterations in CA1 in mild Alzheimer disease and mild cognitive impairment. Neurology.

[6] Callahan LM, Vaules WA, Coleman PD (1999). Quantitative decrease in synaptophysin message expression and increase in cathepsin D message expression in Alzheimer disease neurons containing neurofibrillary tangles. J Neuropathol Exp Neurol.

[7] Callahan LM, Vaules WA, Coleman PD (2002). Progressive reduction of synaptophysin message in single neurons in Alzheimer disease. J Neuropathol Exp Neurol.

[8] Yao PJ (2004). Synaptic frailty and clathrin-mediated synaptic vesicle trafficking in Alzheimer’s disease. Trends Neurosci.

[9] Yao PJ, Zhu M, Pyun EI, Brooks AI, Therianos S, Meyers VE, Coleman PD (2003). Defects in expression of genes related to synaptic vesicle trafficking in frontal cortex of Alzheimer’s disease. Neurobiol Dis.

[10] Sze CI, Bi H, Kleinschmidt-DeMasters BK, Filley CM, Martin LJ (2000). Selective regional loss of exocytotic presynaptic vesicle proteins in Alzheimer’s disease brains. J Neurol Sci.

[11] Shimohama S, Kamiya S, Taniguchi T, Akagawa K, Kimura J (1997). Differential involvement of synaptic vesicle and presynaptic plasma membrane proteins in Alzheimer’s disease. Biochem Biophys Res Commun.

[12] Zhang FX, Sun QJ, Zheng XY, Lin YT, Shang W, Wang AH, Duan RS, Chi ZF (2014). Abnormal expression of synaptophysin, SNAP-25, and synaptotagmin 1 in the hippocampus of kainic acid-exposed rats with behavioral deficits. Cell Mol Neurobiol.

[13] Yoo DY, Jung HY, Kim JW, Yim HS, Kim DW, Nam H, Suh JG, Choi JH, Won MH, Yoon YS, Hwang IK (2016). Reduction of dynamin 1 in the hippocampus of aged mice is associated with the decline in hippocampal-dependent memory. Mol Med Rep.

[14] Shao CY, Mirra SS, Sait HB, Sacktor TC, Sigurdsson EM (2011). Postsynaptic degeneration as revealed by PSD-95 reduction occurs after advanced Aβ and tau pathology in transgenic mouse models of Alzheimer’s disease. Acta Neuropathol.

[15] Lin Z, Gu J, Xiu J, Mi T, Dong J,Tiwari JK (2012). Traditional chinese medicine for senile dementia. Evid Based Complement Alternat Med.

[16] Xia Z, Zhang R, Wu P, Xia Z, Hu Y (2012). Memory defect induced by β-amyloid plus glutamate receptor agonist is alleviated by catalpol and donepezil through different mechanisms. Brain Res.

[17] Xu G, Xiong Z, Yong Y, Wang Z, Ke Z, Xia Z, Hu Y (2010). Catalpol attenuates MPTP induced neuronal degeneration of nigral-striatal dopaminergic pathway in mice through elevating glial cell derived neurotrophic factor in striatum. Neuroscience.

[18] Zhang XL, An LJ, Bao YM, Wang JY, Jiang B (2008). d-galactose administration induces memory loss and energy metabolism disturbance in mice: protective effects of catalpol. Food and chemical toxicology. an international journal published for the British Industrial Biological Research Association.

[19] Jiang B, Shen RF, Bi J, Tian XS, Hinchliffe T, Xia Y (2015). Catalpol: a potential therapeutic for neurodegenerative diseases. Curr Med Chem.

[20] Jiang B, Du J, Liu JH, Bao YM, An LJ (2008). Catalpol attenuates the neurotoxicity induced by beta-amyloid(1-42) in cortical neuron-glia cultures. Brain Res.

[21] Wang JH, Xie H, Zhao TK, Kang B (2015). Catalpol regulates cholinergic nerve system function through effect on choline acetyl-transferase not M receptor affinity. Biomed Pharmacother.

[22] Wang Q, Xing M, Chen W, Zhang J, Qi H, Xu X (2012). HPLC-APCI-MS/MS method for the determination of catalpol in rat plasma and cerebrospinal fluid: application to an in vivo pharmacokinetic study. J Pharm Biomed Anal.

[23] Wang Z, Liu Q, Zhang R, Liu S, Xia Z, Hu Y (2009). Catalpol ameliorates beta amyloid-induced degeneration of cholinergic neurons by elevating brain-derived neurotrophic factors. Neuroscience.

[24] Bramham CR, Messaoudi E (2005). BDNF function in adult synaptic plasticity: the synaptic consolidation hypothesis. Prog Neurobiol.

[25] Cohen-Cory S, Kidane AH, Shirkey NJ, Marshak S (2010). Brain-derived neurotrophic factor and the development of structural neuronal connectivity. Developmental Neurobiology.

[26] Liu J, He QJ, Zou W, Wang HX, Bao YM, Liu YX, An LJ (2006). Catalpol increases hippocampal neuroplasticity and up-regulates PKC and BDNF in the aged rats. Brain Res.

[27] Erickson CA, Barnes CA (2003). The neurobiology of memory changes in normal aging. Exp Gerontol.

[28] Ennaceur A (2010). One-trial object recognition in rats and mice: methodological and theoretical issues. Behav Brain Res.

[29] Findeis MA (2007). The role of amyloid beta peptide 42 in Alzheimer’s disease. Pharmacol Ther.

[30] Roux A, Uyhazi K, Frost A, De Camilli P (2006). GTP-dependent twisting of dynamin implicates constriction and tension in membrane fission. Nature.

[31] Funke L, Dakoji S, Bredt DS (2005). Membrane-associated guanylate kinases regulate adhesion and plasticity at cell junctions. Annu Rev Biochem.

[32] Glantz LA, Gilmore JH, Hamer RM, Lieberman JA, Jarskog LF (2007). Synaptophysin and postsynaptic density protein 95 in the human prefrontal cortex from mid-gestation into early adulthood. Neuroscience.

[33] Park Y, Hernandez JM, van den Bogaart G, Ahmed S, Holt M, Riedel D, Jahn R (2012). Controlling synaptotagmin activity by electrostatic screening. Nat Struct Mol Biol.

[34] Mallozzi C, D’Amore C, Camerini S, Macchia G, Crescenzi M, Petrucci TC, Di Stasi AM (2012). Phosphorylation and nitration of tyrosine residues affect functional properties of Synaptophysin and Dynamin I, two proteins involved in exo-endocytosis of synaptic vesicles. Biochim Biophys Acta.

[35] Lacor PN, Buniel MC, Furlow PW, Clemente AS, Velasco PT, Wood M, Viola KL, Klein WL (2007). Abeta oligomer-induced aberrations in synapse composition, shape, and density provide a molecular basis for loss of connectivity in Alzheimer’s disease. J Neurosci.

[36] Palop JJ, Mucke L (2010). Amyloid-beta-induced neuronal dysfunction in Alzheimer’s disease: from synapses toward neural networks. Nat Neurosci.

[37] Tu S, Okamoto S, Lipton SA, Xu H (2014). Oligomeric Aβ-induced synaptic dysfunction in Alzheimer’s disease. Mol Neurodegener.

[38] Mucke L, Selkoe DJ (2012). Neurotoxicity of amyloid β-protein: synaptic and network dysfunction. Cold Spring Harb Perspect Med.

[39] O’Leary OF, Wu X, Castren E (2009). Chronic fluoxetine treatment increases expression of synaptic proteins in the hippocampus of the ovariectomized rat: role of BDNF signalling. Psychoneuroendocrinology.

[40] Yu W, Lu B (2012). Synapses and dendritic spines as pathogenic targets in Alzheimer’s disease. Neural Plast.

[41] Tanzi RE (2005). The synaptic Abeta hypothesis of Alzheimer disease. Nat Neurosci.

[42] Autry AE, Monteggia LM (2012). Brain-derived neurotrophic factor and neuropsychiatric disorders. Pharmacol Rev.

[43] Frings L, Wagner K, Quiske A, Schwarzwald R, Spreer J, Halsband U, Schulze-Bonhage A (2006). Precuneus is involved in allocentric spatial location encoding and recognition. Exp Brain Res.

[44] Ennaceur A, Michalikova S, Bradford A, Ahmed S (2005). Detailed analysis of the behavior of Lister and Wistar rats in anxiety, object recognition and object location tasks. Behav Brain Res.

[45] Hollup SA, Kjelstrup KG, Hoff J, Moser MB, Moser EI (2001). Impaired recognition of the goal location during spatial navigation in rats with hippocampal lesions. J Neurosci.

[46] Ennaceur A, Neave N, Aggleton JP (1997). Spontaneous object recognition and object location memory in rats: the effects of lesions in the cingulate cortices, the medial prefrontal cortex, the cingulum bundle and the fornix. Exp Brain Res.

[47] Dere E, Huston JP (2007). De Souza Silva MA. The pharmacology, neuroanatomy and neurogenetics of one-trial object recognition in rodents. Neurosci Biobehav Rev.

[48] Ainge JA, Heron-Maxwell C, Theofilas P, Wright P, de Hoz L, Wood ER (2006). The role of the hippocampus in object recognition in rats: examination of the influence of task parameters and lesion size. Behav Brain Res.

[49] Rapp PR, Deroche PS, Mao Y, Burwell RD (2002). Neuron number in the parahippocampal region is preserved in aged rats with spatial learning deficits. Cereb Cortex.

[50] VanGuilder HD, Farley JA, Yan H, Van Kirk CA, Mitschelen M, Sonntag WE, Freeman WM (2011). Hippocampal dysregulation of synaptic plasticity-associated proteins with age-related cognitive decline. Neurobiol Dis.

[51] VanGuilder HD, Yan H, Farley JA, Sonntag WE, Freeman WM (2010). Aging alters the expression of neurotransmission-regulating proteins in the hippocampal synaptoproteome. J Neurochem.

[52] Nicolle MM, Gallagher M, McKinney M (1999). No loss of synaptic proteins in the hippocampus of aged, behaviorally impaired rats. Neurobiol Aging.

[53] Masliah E, Crews L, Hansen L (2006). Synaptic remodeling during aging and in Alzheimer’s disease. J Alzheimers Dis.

[54] Harada A, Teng J, Takei Y, Oguchi K, Hirokawa N (2002). MAP2 is required for dendrite elongation, PKA anchoring in dendrites, and proper PKA signal transduction. J Cell Biol.

[55] Sun MK, Alkon DL (2012). Activation of protein kinase C isozymes for the treatment of dementias. Adv Pharmacol.

[56] Hongpaisan J, Sun MK, Alkon DL (2011). PKC ε activation prevents synaptic loss, Aβ elevation, and cognitive deficits in Alzheimer’s disease transgenic mice. J Neurosci.

[57] Toullec D, Pianetti P, Coste H, Bellevergue P, Grand-Perret T, Ajakane M, Baudet V, Boissin P, Boursier E, Loriolle F, Duhamelll L, Charon D, Kirilovsky J (1991). The bisindolylmaleimide GF 109203X is a potent and selective inhibitor of protein kinase C. J Biol Chem.

[58] Hers I, Tavaré JM, Denton RM (1999). The protein kinase C inhibitors bisindolylmaleimide I (GF 109203x) and IX (Ro 31-8220) are potent inhibitors of glycogen synthase kinase-3 activity. FEBS Lett.

[59] Greco SJ, Bryan KJ, Sarkar S, Zhu X, Smith MA, Ashford JW, Johnston JM, Tezapsidis N, Casadesus G (2010). Leptin reduces pathology and improves memory in a transgenic mouse model of Alzheimer’s disease. J Alzheimers Dis.

[60] Hu H, Zhang R, Zhang Y, Xia Z, Hu Y (2010). Role of CREB in the regulatory action of sarsasapogenin on muscarinic M1 receptor density during cell aging. FEBS Lett.

