# Supplementary material for: Catalpol protects synaptic proteins from beta-amyloid induced neuron injury and improves cognitive functions in aged rats
Source: Oncotarget. 2017 May 17;8(41):69303–15. doi: 10.18632/oncotarget.17951 (PMC5642480; doi:10.18632/oncotarget.17951)
Supplement: Supplementary file 1 [file oncotarget-08-69303-s001.pdf]

Catalpol protects synaptic proteins from beta-amyloid induced neuron injury and improves cognitive functions in aged rats

SUPPLEMENTARY MATERIALS

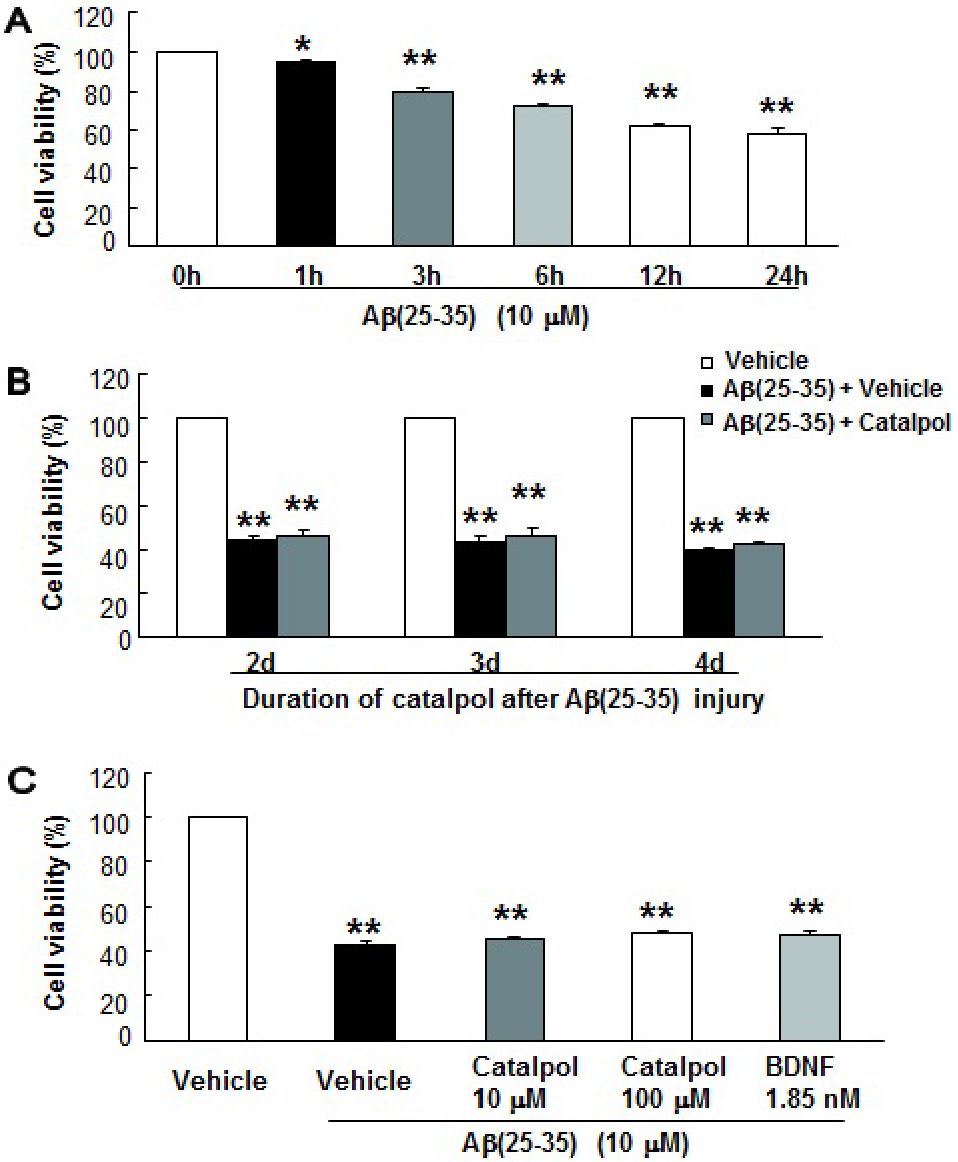

Supplementary Figure 1: Effects of catalpol (10 µM) on cell survival of the primary cortical neurons injured by Aβ. (A) Effects of $A\beta_{25-35}$ on the viability of primary cortical neurons after different exposure durations. (B) The effects of catalpol on the viability of $A\beta_{25-35}$-treated neurons after different duration. (C) The effects of catalpol on the viability of $A\beta_{25-35}$-treated cortical neurons at different doses. All values are normalized to the control group values and expressed as the mean ± SEM of 4 independent experiments. * and ** indicate $p<0.05$ and $p<0.01$, respectively, compared with the corresponding control group.

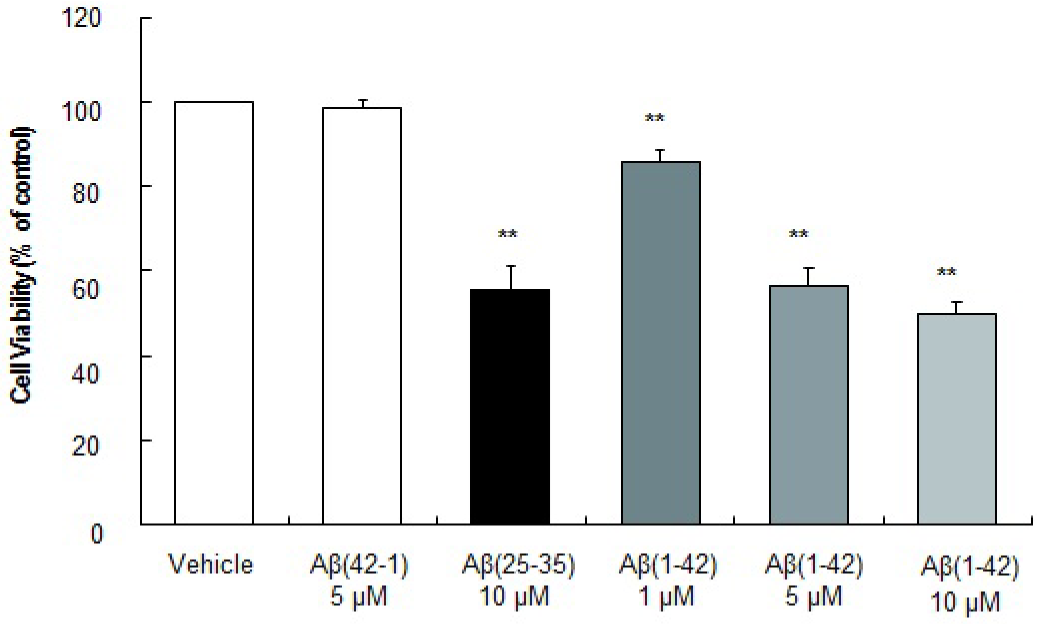

Supplementary Figure 2: Effects of $A\beta_{1-42}$ on the viability of primary cortical neurons after different exposure durations. $A\beta_{42-1}$, $A\beta_{25-35}$ and $A\beta_{1-42}$ were added respectively for 12 h followed by MTT assay. The results are normalized to the control group and expressed as the mean ± SEM of 4 independent experiments. * and ** indicate $p<0.05$ and $p<0.01$, respectively, compared with the corresponding control group.
